# The Virtual Client Experience Survey for Mental Health and Addictions: Revalidation of a Survey to Measure Client and Family Experiences of Virtual Care

**DOI:** 10.2196/49844

**Published:** 2025-01-03

**Authors:** Allison Crawford, Anne Kirvan, Marcos Sanches, Amanda Gambin, Denise Canso, Eva Serhal

**Affiliations:** 1 Centre for Addiction and Mental Health Toronto, ON Canada

**Keywords:** virtual care, digital health, mental health, client satisfaction, health care quality, Virtual Client Experience Survey, telehealth, telemedicine, eHealth

## Abstract

**Background:**

The onset of the COVID-19 pandemic precipitated a rapid shift to virtual care in health care settings, inclusive of mental health care. Understanding clients’ perspectives on virtual mental health care quality will be critical to informing future policies and practices.

**Objective:**

This study aimed to outline the process of redesigning and validating the Virtual Client Experience Survey (VCES), which can be used to evaluate client and family experiences of virtual care, specifically virtual mental health and addiction care.

**Methods:**

The VCES was adapted from a previously validated telepsychiatry survey. All items were reviewed and updated, with particular attention to the need to ensure relevance across mental health care sectors and settings. The survey was then revalidated using the 6 domains of health care quality of the Institute of Medicine (IOM) as a guiding framework. These 6 domains include being safe, effective, patient-centered, efficient, timely, and equitable. The VCES was piloted with a convenience sample of clients and family members accessing outpatient care at the Centre for Addiction and Mental Health (CAMH) in Toronto, Ontario, through video or telephone. A confirmatory factor analysis (CFA) was conducted in MPlus and used to test the factorial structures of the VCES, with minor respecification of the model based on modification indices, factor loadings, reliability, and item-total correlation. The respecifications were checked for alignment with the construct definitions and item interpretation. The reliability of the constructs was estimated by the Cronbach α coefficient.

**Results:**

The survey was completed 181 times. The construct reliability was generally high. Timely was the only subscale with an α lower than 0.7; all others were above 0.8. In all cases, the corrected item-total correlation was higher than 0.3. For the CFA, the model was adjusted after multiple imputations with 20 datasets. The mean chi-square value was 437.5, with *df*=199 (*P*<.001). The mean root mean square error of approximation (RMSEA) was 0.08 (SD 0.002), the mean confirmatory fit index (CFI) was 0.987 (SD 0.001), the mean Tucker-Lewis Index (TLI) was 0.985 (SD 0.001), and the mean standardized root mean square residual (SRMR) was 0.04 (SD 0.001).

**Conclusions:**

This study describes the validation of the VCES to evaluate client and family experiences of virtual mental health and addictions care. Given the widespread uptake of virtual care, this survey has broad applicability across settings that provide mental health and addiction care. The VCES can be used to guide targeted quality improvement initiatives across health care quality domains. By effectively addressing challenges as they emerge, it is anticipated that we will continue to move toward hybrid modalities of practice that leverage the strengths and benefits of telephone, video, and in-person care to effectively respond to unique client and family needs and circumstances.

## Introduction

The onset of the COVID-19 pandemic precipitated a rapid shift to virtual care in health care settings, inclusive of mental health care. Virtual care, often referred to as telehealth or telemedicine, refers to the provision of clinical care using information and communication technologies and can be delivered synchronously (ie, in real-time, at a distance) or asynchronously (ie, separated by time and space) [1,2]. During the early phases of the pandemic, synchronous forms of virtual care, such as video- and telephone-based care, replaced in-person care as the leading modalities of practice in many health care settings [3-6].

Research evaluating virtual mental health care has consistently shown high levels of both client satisfaction [3-7] and provider satisfaction [5,8]. However, recent literature also highlights challenges with virtual care, including inequities in access to and engagement with virtual care [9,10], technology challenges [11], lack of connection between clients and health care providers [4], and concerns regarding privacy and safety [12]. Understanding clients’ perspectives on virtual mental health care quality will be critical to inform future policies and practices. In addition, it will be important to understand how these experiences vary across sociodemographic groups, particularly in light of long-standing inequities in health care access and outcomes that risk being reinforced by the widespread uptake of virtual care [10].

To date, there is only 1 known validated measure of client experiences of virtual mental health care, specifically telepsychiatry [6], and there are no known validated measures that can be used to evaluate client experiences across varied types of mental health care. Since the transition to virtual care, a majority of patients across multiple surveys indicate a preference for virtual care to continue to exist as an option [3,4], with many preferring a hybrid approach that combines both in-person and virtual modalities [3]. Validated means to understand client and family experiences of care will be critical to inform evidence-based decision-making regarding virtual care and hybrid modalities of practice moving forward.

This paper outlines the process to redesign and validate the Virtual Client Experience Survey (VCES) that can be used to evaluate client and family experiences of virtual mental health and addictions care.

## Methods

### Overview

At the outset of the COVID-19 pandemic, the VCES was adapted from a previously validated telepsychiatry survey developed and used within the TeleMental Health program at the Centre for Addiction and Mental Health (CAMH), a mental health and addiction hospital located in Toronto, Ontario [6]. This telepsychiatry survey was updated to respond to the pressing need to evaluate virtual care both within our hospital and externally. Several adaptations were made to extend the use of the tool beyond its original intended use with clients accessing psychiatric consultation and assessment through videoconference [6].

### Survey Redesign

The approach to developing and validating the original telepsychiatry survey is outlined in the paper by Serhal et al [6]. Our team of clinicians, researchers, and health care leaders reviewed the telepsychiatry survey and adapted it for use in the current context. All items were reviewed and updated, in particular, with the need to ensure relevance that is accorded with the expansion of virtual care provision beyond physicians and psychiatrists. For example, items on the newly developed VCES ask about the client’s experience with the virtual appointment instead of the telepsychiatry appointment. Another item asks whether the health care provider explained the risks and benefits of treatments or interventions rather than the risks and benefits of medications. After consultation with clinicians across multiple programs and services at the hospital, the survey was also updated to ensure applicability not only to clients but to family members as well. This was an important shift given the key role that families play in mental health and addiction care and recovery [13,14] and in supporting access to care through videoconference.

The transition to virtual care during the COVID-19 pandemic also necessitated the examination of virtual care from a digital health equity perspective [10]. Our team consulted with the CAMH Health Equity Office as well as the Provincial System Support Program (PSSP), which administers the Ontario Perception of Care Tool for Mental Health and Addictions (OPOC-MHA) [15], regarding the addition of sociodemographic questions to facilitate health equity analyses. Furthermore, 5 items from the OPOC-MHA were also added to facilitate alignment and comparison between the 2 tools.

The VCES sociodemographic questions ask about gender, age, geographic region, whether the client was born in Canada, racial or ethnic group, illness and disability, and other factors. Clients and families were also asked about their comfort with technology, where they accessed the virtual care appointment from (eg, home, health care organization), the type of device they used to access virtual care (eg, computer, smartphone), and which videoconference platform was used. The telephone was included as an option on the survey, as CAMH, like many health care settings, began providing more services by telephone during the pandemic, particularly for individuals experiencing barriers to video-based care. Finally, as recommended by Serhal et al [6], we added additional survey items to assess access to care, and physical and emotional safety during virtual care appointments. We also added an item about compassionate care, given our research group’s interest in compassion as a critical factor in the therapeutic relationship and inpatient and family experience of care [16,17].

The redesigned VCES consists of 22 items to assess the overall quality of care. These items are rated on a 4-point Likert scale (1=strongly disagree, 2=disagree, 3=agree, 4=strongly agree) with additional options including “not applicable” and “prefer not to answer.” The final question on the survey is an open-text question to elicit any additional feedback.

### Conceptual Framework

Following the adaption of the survey, the survey was revalidated using the 6 domains of health care quality of the Institute of Medicine (IOM) as a guiding framework. These 6 domains include being safe, effective, patient-centered, efficient, timely, and equitable [18]. The IOM domains were selected as a guiding framework to provide a comprehensive evaluation of client satisfaction and experience and to guide targeted quality improvement efforts across the different domains. The heath quality domain definitions [18] have been adapted in Table 1 below for the VCES.

**Table 1:** Health quality domain definitions.

Health quality domains	Original definition	Adapted definition for the VCES^a^ domains
Safe	“Avoiding injuries to patients from the care that is intended to help them [18].”	Virtual care that is physically and psychologically safe and minimizes potential risks.
Effective	“Providing services based on scientific knowledge to all who could benefit and refraining from providing services to those not likely to benefit (avoiding underuse and overuse)” [18]	Effective delivery of virtual care, inclusive of the use of virtual care technologies, to facilitate engagement in virtual care.
Patient-centered	“Providing care that is respectful of and responsive to individual patient preferences, needs, and values and ensuring that patient values guide all clinical decisions” [18]	Virtual care that is collaborative, respectful, and responsive to client and family preferences, needs, and values.
Efficient	“Avoiding waste, in particular, waste of equipment, supplies, ideas, and energy” [18]	Efficiency and ease in accessing virtual care.
Timely	“Reducing waits and sometimes harmful delays for both those who receive and those who give care” [18]	Timely access to virtual care for clients and families, including limited wait times for services.
Equitable	“Providing care that does not vary in quality because of personal characteristics such as gender, ethnicity, geographic location, and socioeconomic status” [18]	Virtual care that does not vary in quality based on personal characteristics and sociodemographic factors.

^a^VCES: Virtual Client Experience Survey.

### Question Validation

#### Survey Dissemination

The VCES was piloted with a convenience sample of clients (16 years of age and older) and family members accessing a wide range of outpatient mental health and addiction services at CAMH through video or telephone. Survey data was collected using Research Electronic Data Capture (REDCap), a web-based application for securely collecting surveys and managing data [19,20]. The electronic survey took approximately 15 minutes to complete. Survey completion was voluntary, and all survey responses were anonymous. Survey data collection took place in 2021.

Information about the VCES and the process for survey administration was shared with clinicians and administrative staff using multiple channels, including the organizational intranet, email, support and sponsorship from clinical program directors, and program-level presentations by the project team. CAMH clinicians and administrative staff were asked to include information about the VCES, including the REDCap survey links, by email to all CAMH clients and families before their virtual care appointment. A template script for email communication to clients was provided, including a reminder that survey completion was voluntary and that all responses were anonymous. Clients and families were asked to complete a survey once per program or service that they participated in. The survey was made available to clients seeing providers across programs at the hospital, which includes multidisciplinary care providers (eg, physicians, nurses, social workers, occupational therapists, psychologists).

#### Content Validity

The 22 Likert-scale items on the VCES were independently reviewed by a panel of 5 individuals. including clinicians, researchers, and health care leaders, to determine alignment with the following IOM health quality domains: safe, effective, patient-centered, efficient, and timely [18]. Equity, the sixth IOM domain, is assessed through a comparison of survey response options by sociodemographic group. The panel then met through videoconference to discuss discrepancies until a consensus was reached.

#### Factorial Structure

To test the factorial structure of the VCES, a confirmatory factor analysis (CFA) was adjusted in Mplus (version 8.2; Muthén & Muthén) [21] using the method of weighted least squares with mean and variance adjusted chi-square test (WLSMV), which is recommended for ordinal outcomes [22]. Multiple imputation using the covariance method [23], with 20 imputed datasets, was used to handle missing values, which were present in 45% of the surveys, with 80% of surveys having 3 or fewer items missing. The evaluation of the model relied on the fit indices RMSEA (root means square error of approximation) [24], CFI (comparative fit index) [25], TLI (Tucker-Lewis index) [26], and SRMR (standardized root mean square residual) [27], as well as the inspection of standardized factor loadings, variance explained, and modification index relative to each item. The initial factorial structure was defined following expert opinion, with minor respecification of the model based on modification indices, factor loadings, reliability, and item-total correlation. The respecifications were checked for alignment with the construct definitions and item interpretation. Cutoffs for fit indices in CFA and reliability are not universally accepted [28]. That said, based on results from Hu and Bentler [29], TLI and CFI higher than 0.95, SRMR lower than 0.08, and RMSEA lower than 0.06 are considered acceptable.

#### Reliability

The reliability of the constructs was estimated by the Cronbach α coefficient [30], a measure of internal consistency between the items. The corrected item-total correlation (correlation between the item and the total scale score with the item removed) and the α coefficient for the scale with the item removed were also used as sources of information as to how the items fit the factors. A Cronbach α of 0.7 or higher is considered good [28].

### Ethical Considerations

Consent to participate was implied. The survey contained an introductory section explaining the purpose of the survey, informing potential respondents that participation was voluntary and anonymous, and informing them that the findings would be presented as grouped or aggregated data for any presentation or publication. There was no compensation offered to clients and families for completing the survey. This project received ethical approval from CAMH Quality Projects Ethics Review (QPER-2021-000).

## Results

### Descriptive Analysis

In total, the survey was completed 181 times, with 99 surveys completed by clients, 16 by support persons on behalf of clients, and 22 by family members or caregivers. Respondent information is missing on 44 surveys. Sociodemographic characteristics are included in Table 2.

Most respondents used a computer (113/179, 62.4%) or tablet or smartphone (58/179, 32%). Only 5.5% (10/179) reported using a telephone (audio only). The majority of respondents were either comfortable with technology (64/179, 35.8%) or very comfortable (85/179, 47.5%).

**Table 2:** Participant characteristics.

Characteristics		Participants, n (%)
**Role**		
	Registered CAMH^a^ client	99 (72.3)
	Supporting a registered CAMH client	16 (11.7)
	Support person	22 (16.1)
**Sex**		
	Female	95 (52.5)
	Male	76 (42)
	Transgender: female to male	3 (1.7)
	Transgender: male to female	2 (1.1)
	Other	4 (2.2)
	Do not know	1 (0.6)
**Age group**		
	13-18 years	7 (3.9)
	19-25 years	52 (28.7)
	26-34 years	59 (32.6)
	35-44 years	17 (9.4)
	45-54 years	24 (13.3)
	55-64 years	19 (10.5)
	65+	3 (1.7)
**Born in Canada**		
	Yes	121 (67.2)
	No	59 (32.6)
	Prefer not to answer	1 (0.6)
**Ethnicity**		
	Asian: East (eg, Chinese, Japanese, Korean)	8 (4.4)
	Asian: South (eg, Indian, Pakistani, Sri Lankan)	18 (9.9)
	Asian: South East (eg, Malaysian, Filipino, Vietnamese)	4 (2.2)
	Black: African (e.g., Ghanaian, Kenyan, Somali)	9 (5)
	Black: Caribbean (eg, Barbadian, Jamaican)	5 (2.8)
	Black: North American (eg, Canadian, American)	2 (1.1)
	First Nations	2 (1.1)
	Indian: Caribbean (eg, Guyanese with origins in India)	1 (0.6)
	Latin American (eg, Argentinean, Chilean, Salvadoran)	7 (3.9)
	Métis	1 (0.6)
	Middle Eastern (eg, Egyptian, Iranian, Lebanese)	9 (5)
	White: European (eg, English, Italian, Portuguese, Russian)	29 (16)
	White: North American (eg, Canadian, American)	73 (40.3)
	Mixed heritage (eg, Black-African and White-North American)	6 (3.3)
	Other(s)	3 (1.7)
	Prefer not to answer	4 (2.2)
**Illness and disability**		
	Chronic illness	24 (13.3)
	Developmental disability	25 (13.8)
	Substance use disorder	21 (11.6)
	Learning disability	28 (15.5)
	Mental illness	116 (65.1)
	Physical disability	7 (3.9)
	Sensorial disability	3 (1.7)
	None	30 (16.6)
	Others	6 (3.3)
	Prefer not to answer	14 (7.7)

^a^CAMH: Centre for Addiction and Mental Health.

### Reliability

The construct reliability, as measured by Cronbach α, was generally high. Timely was the only subscale with an α lower than 0.7; all others were above 0.8 (more details in Table 3). In all cases, the corrected item-total correlation was higher than 0.3. Considering the items in the model, item 9 (effective) and items 2 and 10 (timely) had lower corrected item-total correlation, with all the other items having correlations around 0.7 or above. Within the “timely” factor, the low item-total correlation was likely reflective of the small number of items associated with this domain.

**Table 3:** Confirmatory factor analysis results and item statistics.

Factors and survey items			Loading^a^		SE^b^		Missing^c^, %		*R* ^2 d^		Correlation^e^		Mean^f^ (SD^g^)
**Efficient (Cronbach α^h^>=0.87)**													
	1. It was easy to access virtual care at CAMH^i^.	0.88		0.03		12		0.78		0.79		3.47 (0.71)	
	3. It was easy to book my virtual appointment.	1.00		0.02		9		1.00		0.85		3.51 (0.66)	
	21. The physical location of where I accessed my virtual appointment was convenient for me.	0.88		0.03		8		0.78		0.72		3.54 (0.72)	
**Timely (Cronbach α=0.66)**													
	2. The wait time for services was reasonable for me.^j^	0.72		0.06		23		0.52		0.46		3.26 (0.82)	
	10. I was able to get a virtual appointment sooner than an in-person healthcare appointment.	0.81		0.06		42		0.66		0.46		3.27 (0.76)	
**Effective (Cronbach α=0.80)**													
	4. During my virtual appointment, I was able to see the healthcare provider clearly.	0.89		0.03		10		0.79		0.69		3.54 (0.70)	
	5. During my virtual appointment, I was able to hear the healthcare provider clearly.	0.91		0.02		6		0.84		0.69		3.43 (0.75)	
	9. I believe virtual care is just as effective as in-person healthcare.	0.55		0.06		15		0.31		0.38		2.99 (0.89)	
	22. Overall, I am satisfied with my virtual appointment.	0.96		0.02		9		0.92		0.74		3.51 (0.72)	
**Patient-centered (Cronbach α=0.96)**													
	6. I am confident that the healthcare provider at CAMH and my other service providers are working as a team.	0.85		0.03		10		0.72		0.77		3.48 (0.73)	
	7. I feel that there was an adequate amount of time allotted for the virtual appointment.	0.87		0.03		5		0.75		0.79		3.54 (0.68)	
	8. I felt comfortable during my virtual appointment.	0.92		0.02		13		0.85		0.81		3.50 (0.77)	
	12. Staff understood and responded to my needs and concerns.^j^	0.95		0.01		16		0.89		0.83		3.52 (0.72)	
	13. I was treated with respect by program staff.^j^	0.96		0.01		7		0.92		0.89		3.62 (0.71)	
	14. I received compassionate virtual care.	0.97		0.01		15		0.94		0.91		3.59 (0.73)	
	16. The healthcare provider spoke with me about my mental health and/or addiction in a way that I could understand.	0.97		0.01		27		0.95		0.89		3.54 (0.73)	
	17. I was involved as much as I wanted to be in decisions about my treatment services and supports.^j^	0.97		0.01		29		0.94		0.89		3.54 (0.69)	
	19. I am confident that I will be able to follow the healthcare provider’s recommendations.	0.87		0.03		16		0.75		0.75		3.36 (0.76)	
**Safe (Cronbach α=0.89)**													
	11. I was assured my personal information was kept confidential.^j^	0.87		0.03		10		0.76		0.80		3.50 (0.74)	
	15. I felt safe (emotionally and physically) during my virtual appointment.	0.99		0.01		8		0.98		0.82		3.56 (0.74)	
	18. The healthcare provider explained to me the benefits and risks of any treatments or interventions that were recommended during my virtual appointment.	0.89		0.02		15		0.79		0.79		3.45 (0.76)	
	20. I understand what to do if I have a mental health and/or addiction emergency following this appointment.	0.82		0.03		11		0.67		0.69		3.44 (0.73)	

^a^Confirmatory factor analysis standardized loadings.

^b^Standard error of the confirmatory factor analysis loading.

^c^Proportion of the subject with missing values in the item. These items were imputed using multiple imputation for the confirmatory factor analysis analysis.

^d^Variance explained by the confirmatory factor analysis model.

^e^Corrected item-total correlation (correlation between the item and the sum score with the item removed).

^f^Item mean (Item values range from 1=strongly disagree to 4=strongly disagree).

^g^Item standard deviation.

^h^Cronbach α (internal consistency).

^i^CAMH is referenced in the survey that was disseminated internally. The external version refers to “this organization.”

^j^Items from the Ontario Perception of Care Tool for Mental Health and Addictions (OPOC-MHA) [15].

### Confirmatory Factor Analysis

Confirmatory Factor Analysis loadings are shown in Table 3 above. The model was adjusted after multiple imputations with 20 datasets. We show fit statistics using the mean and SD of these 20 datasets. The mean chi-square value was 437.5, with *df*=199 (*P*<.001). The mean RMSEA was 0.08 (SD 0.002), which is considered reasonable. The mean CFI was 0.987 (SD 0.001), the mean TLI was 0.985 (SD 0.001), and the mean SRMR was 0.04 (SD 0.001). These values are considered to be quite good. With the exception of item 9 in the “effective” factor, all the others have standardized loadings higher than 0.7.

The model shown in Table 3 had 2 respecifications: item 19 was moved from “effective” to “patient-centered,” and item 18 was moved from “patient-centered” to “safe.” These changes were based on the modification index and discussions with the project team; however, they did not meaningfully change the fit measures. While item 9 does not fit as well in the “effective factor,” it was maintained as is, as it represented the best conceptual fit.

The sample size for structural equation models is not well defined because of the high number of parameters of interest and broad range of model complexity. Considering our model with 5 factors with an average of 4 indicators per factor and loadings around 0.8, Wolf et al [31] found that a sample size of 120 tends to be sufficient for power above 80%. These results are negatively affected by the presence of missing values; as such, our sample of 181 is expected to have reasonable power for a CFA analysis.

## Discussion

### Principal Findings

This paper provides a valid and reliable measure to evaluate client and family experiences of virtual mental health and addiction care, focusing on 6 domains of health care quality. This survey has been used internally at CAMH to guide the next steps and recommendations for virtual mental health care and has been shared externally in both English and French to support quality measurement within other organizations. Information about the survey was shared through a webinar, and the survey was subsequently downloaded hundreds of times across Canada and internationally.

### Implications for Virtual Mental Health Care

Given the widespread uptake of virtual care, this survey has broad applicability across settings that provide mental health and addiction care. The VCES can be used to guide targeted quality improvement initiatives across the 6 IOM domains of health care quality, including safe, effective, patient-centered, efficient, timely, and equitable care [18]. It also has the potential to be adapted to other health care specialties and contexts.

### Considerations for Administering the VCES

From our experience, we found the use of REDCap (Research Electronic Data Capture; Vanderbilt University) to be an effective means to disseminate the survey, collate results, and ensure anonymity. Some training and guidance regarding survey administration processes were required to ensure that the survey was delivered as part of the standard of care. We found it advantageous to delineate a time-limited window for survey dissemination and completion to limit time demands on both clients and clinicians. In terms of interpreting results, we set a cutoff of 3 (agree) and above as a positive result indicating high quality of care. Finally, there was a high degree of interest in receiving feedback on the survey results. We offered aggregated program-level reports once an adequate sample of surveys was completed in order to preserve respondent anonymity; this is particularly important as demographic questions attached to the survey could render individual results identifiable.

### Next Steps

While the findings from the VCES provided invaluable and timely feedback, it is important not to limit patient and family feedback to a survey. The open text option on the survey provided a breadth of feedback not necessarily addressed within the existing survey questions. Analysis of this qualitative data may spur future adaptations to the survey and, on a practical level, may steer quality improvement initiatives resulting from survey findings. Furthermore, ongoing patient and family engagement in interpreting survey results and prioritizing and guiding quality improvement will be essential to making meaningful and impactful improvements.

Patient experience of virtual care should also be considered alongside provider experience of virtual care. To complement findings from the VCES and to capture another key perspective within health care, our team has developed and validated the Virtual Provider Experience Survey (VPES). These complementary tools provide key metrics to inform how health care organizations and systems can move toward the “quadruple aim,” a well-recognized framework for health care that seeks to improve population health outcomes, reduce health care costs, and improve patient, family, and provider experiences of care [32]. The consistent use of standardized tools across settings is a valuable way to inform policy and program planning with respect to virtual care [33] and to further our advancement toward the quadruple aim. Further practice-based research is also needed to examine the relationship between the experience of care and health outcomes for clients and families who access virtual mental health and addiction care and the cost-effectiveness of those services.

### Limitations

While the VCES was validated with 181 survey responses, it is possible that some clients or family members completed the survey more than once if they were involved in multiple programs or services during the evaluation period. In addition, when administering surveys, there is always the risk of self-selection and response bias. We sought to mitigate this risk by asking clinicians and administrative staff to send the survey link to all clients and families accessing virtual care at CAMH. A response rate can be beneficial to ascertain the extent of potential bias; however, we were unable to determine the response rate, as it is unknown how many clients and families received the link in their email correspondence from clinicians or administrative staff. It is quite likely that those who have a higher degree of familiarity or comfort with virtual care were more likely to complete an online survey sent by email.

### Conclusion

We sought to address a notable gap in the literature by redesigning and validating a measure of client and family experiences of virtual mental health care. Client and family perspectives are needed to evaluate the current state of mental health service delivery and highlight areas that need improvement. By effectively addressing challenges as they emerge, it is anticipated that we will continue to move toward hybrid modalities of practice that leverage the strengths and benefits of telephone, video, and in-person care to effectively respond to unique client and family needs and circumstances [11].

## Abbreviations

CAMH: Centre for Addiction and Mental Health
CFA: confirmatory factor analysis
CFI: confirmatory fit index
IOM: Institute of Medicine
OPOC-MHA: Ontario Perception of Care Tool for Mental Health and Addictions
PSSP: Provincial System Support Program
REDCap: Research Electronic Data Capture
RMSEA: root mean square error of approximation
SE: standard error
SRMR: standardized root mean square residual
TLI: Tucker-Lewis index
VCES: Virtual Client Experience Survey
VPES: Virtual Provider Experience Survey
WLSMV: weighted least squares with mean and variance adjusted chi-square test

## Data Availability

The data that support the findings presented in this report are available upon request from the corresponding author.
